# A cancer-unique glycan: de-N-acetyl polysialic acid (dPSA) linked to cell surface nucleolin depends on re-expression of the fetal polysialyltransferase *ST8SIA2* gene

**DOI:** 10.1186/s13046-021-02099-y

**Published:** 2021-09-20

**Authors:** Gregory R. Moe, Lindsay M. Steirer, Joshua A. Lee, Adarsha Shivakumar, Alejandro D. Bolanos

**Affiliations:** UCSF Benioff Children’s Hospital Oakland, 5700 Martin Luther King Jr. Way, Oakland, CA 94609 USA

**Keywords:** Glycans, de-N-acetyl polysialic acid, Nucleolin, ST8SIA2, Immune shielding, cancer

## Abstract

**Background:**

Polysialic acid (polySia) modifies six cell surface proteins in humans mainly during fetal development and some blood cells in adults. Two genes in humans, *ST8SIA2* and *ST8SIA4*, code for polysialyltransferases that synthesize polySia. *ST8SIA2* is highly expressed during fetal development and in cancer but not in adult normal human cells. *ST8SIA4* is expressed in fetal and adult brain, spleen, thymus, and peripheral blood leukocytes and in cancer. We identified a derivative of polySia containing de-N-acetyl neuraminic acid residues (dPSA), which is expressed on the cell surface of human cancer cell lines and tumors but not normal cells.

**Methods:**

dPSA-modified proteins in several human cancer cell lines and normal blood cells were identified using co-immunoprecipitation with anti-dPSA antibodies, mass spectroscopy and Western blot. RNAi and CRISPR were used to knockdown and knockout, respectively, the polysialyltransferase genes in human melanoma SK-MEL-28 and neuroblastoma CHP-134 cell lines, respectively, to determine the effect on production of cell surface dPSA measured by flow cytometry and fluorescence microscopy.

**Results:**

We found that dPSA is linked to or associated with nucleolin, a nuclear protein reported to be on the cell surface of cancer but not normal cells. Knocking down expression of *ST8SIA2* with RNAi or knocking out each gene individually and in combination using CRISPR showed that cell surface dPSA depended on expression of *ST8SIA2*.

**Conclusions:**

The presence of dPSA specifically in a broad range of human cancers but not human adult normal cells offers novel possibilities for diagnosis, prevention and treatment targeting the dPSA antigen that appears to be cancer-specific, consistent across not only human cancers but also species, and may be an unrecognized mechanism of immune shielding.

**Supplementary Information:**

The online version contains supplementary material available at 10.1186/s13046-021-02099-y.

## Background

Human polysialic acid (polySia), is a developmentally regulated homopolymer of α2–8-linked 5-N-acetyl neuraminic acid residues that can be greater than 100 residues in length. Humans have two genes, *ST8SIA2* and *ST8SIA4*, that code for enzymes that synthesize polySia (polysialyltransferases ST8SIA2 and ST8SIA4, respectively). While both genes are highly expressed in humans during fetal development [1], *ST8SIA4* is expressed mainly in lymphoid tissues and lymphocytes [2], while *ST8SIA2* does not appear to be present at significant levels in any adult normal tissues based on Northern blot [1] and publicly available RNA-seq and protein databases (as summarized, for example, by GeneCards: the human gene database [3]). Of the six proteins confirmed to be polysialylated in humans [4–9], neural cell adhesion molecule (NCAM) is the most abundant, particularly during fetal development, and is the most thoroughly investigated [10]. A number of human cancers are reported to express polySia-NCAM abnormally [11–14] where its role in mediating interactions among cells and between cells and the extracellular matrix is associated with metastasis and poor clinical prognosis [11, 14]. Although both polysialyltransferases ST8SIA2 and ST8SIA4 appear to synthesize the same polysaccharide, there may be differences in substrate specificity [15, 16] or functional activity. ST8SIA2 was reported to produce shorter polymers compared to ST8SIA4, ST8SIAII exclusively polysialylates SynCAM1 in mice [16] and, in some circumstances, the two enzymes may work synergistically [17]. Recently, mutations in the promoter region of *ST8SIA2* were associated with schizophrenia [18] suggesting that the functional effect of each enzyme is different even though they produce the same polysaccharide. However, the functional distinctions between the two polysialyltransferases remain unclear.

Previously, we reported the discovery of a de-N-acetylated form of polySia (dPSA) and of anti-dPSA antibodies that were reactive with dPSA antigens [19, 20] on placental trophoblasts, the surface of cancer cells, and inside cancer cells and some normal cells within the perinuclear space [21, 22]. Also, human microbial pathogens that produce polySia, including *Neisseria meningitidis* serogroup B [19] and *Leishmania major* [23], display cell surface dPSA under conditions corresponding to encountering a human host [23, 24]. Although de-N-acetyl sialic acid-containing derivatives of gangliosides have been described previously in human melanoma cell lines [25, 26] and de-N-acetylation of polysaccharides occurs in many species [27], proteins modified with dPSA and the function of dPSA in human cell biology, particularly cancer, are unknown.

In this study, we identified nucleolin as the protein modified or associated with dPSA. We investigated the role of *ST8SIA2* and *ST8SIA4* related to the production of dPSA on the surface of cancer cells. Also, we characterized the effect of interfering with *ST8SIA2* and *ST8SIA4* expression on cell surface dPSA and SK-MEL-28 cell morphology. We found that cell surface dPSA depended on *ST8SIA2* expression. Importantly, we show that cell surface dPSA is unique to cancer cells and widely expressed among different cancers. The unique presence of dPSA on the surface of human trophoblasts, cancer cells, and microbial pathogens suggests the possibility of a role for dPSA in immune shielding since that is a survival feature each has in common.

## Methods

### Antibody reagents

Anti-nucleolin monoclonal mouse antibody MS-3 was acquired from Santa Cruz Biotechnology (Santa Cruz, CA). Irrelevant murine and human subclass control antibodies were obtained from Southern Biotech (Birmingham, AL) and BioXCell (Lebanon, NH). Anti-dPSA monoclonal antibodies (mAbs) SEAM 2 and SEAM 3 and anti-polySia mAb SEAM 12 were produced as described previously [28]. Recombinant SEAM 3 used in some experiments was produced by WuXi AppTec, (Shanghi, China). Irrelevant mAb, 14C7 (murine IgG3), was produced as described previously [29]. Full length recombinant nucleolin produced in HEK293 cells was obtained from OriGene (Rockville, MD). All anti-mouse secondary antibodies conjugated with Alexa Fluor fluorochromes were obtained from Thermo Fisher Scientific (Waltham, MA). SEAM 2, SEAM 3, SEAM 12 and 14C7 used in this study were purified by Protein A affinity chromatography. Recombinant SEAM 3 was additionally purified by size exclusion chromatography. Antibody concentrations were determined by absorbance at 280 nm.

### Cell culture

SK-MEL-28 human melanoma and panels of gastric, ovarian, and pancreatic cancer cell lines (HTB-72, TCP-1008, TCP-1021, and TCP-1026, respectively) were obtained from American Type Culture Collection (ATCC, Manassas, VA) and cultured in medium recommended by ATCC. CHP-134 cells were obtained from MilliporeSigma (Burlington, MA). Kelly cells were a gift from J. Saba at UCSF Benioff Children’s Hospital Oakland. Kelly, SK-MEL-28, RNAi mutant SK-MEL-28, CHP-134, and CRISPR knockout cells were grown routinely in flasks containing RPMI 1640 medium, penicillin/streptomycin, and 10% fetal bovine serum (FBS) at 37 °C in 5% CO_2_. Confluent cells were sub-cultured (1∶3 to 1∶8) by treating with 0.25% (weight/volume) trypsin/0.53 mM EDTA or Accutase® solution (Thermo Fisher Scientific) and washing in media before re-seeding into new growth medium. CHP-134 and CHP-134 CRISPR knockout clone cells were suspended by pipetting. Medium for the SK-MEL-28 mutant cell lines also contained 10 μg/mL blasticidin. Adherent cells used for immunoprecipitation, fluorescence activated cell sorting (FACS) binding, and fluorescence labeling experiments were treated with Accutase® and washed with medium before use. Human normal peripheral blood mononuclear cells (PBMCs) were purchased from AllCells (Alameda, CA).

### Protein extraction from cells

Cells from each cell culture were extracted using the ProteoExtract® Subcellular Proteome Extraction Kit (MilliporeSigma). In brief, the differential detergent extraction procedure used four extraction buffers sequentially, along with a protease inhibitor cocktail to prevent protein degradation during the extraction and Benzonase® nuclease (Sigma-Aldrich) to degrade contaminating nucleic acids. The manufacturer’s instructions for extraction were followed, and the cell extracts were separated into four fractions: F1 (cytosolic fraction), F2 (cell membrane fraction), F3 (nucleic protein fraction), and F4 (cytoskeletal fraction).

### Co-immunoprecipitation

Dynabeads M-270 epoxy magnetic beads (Thermo Fisher Scientific) covalently linked to SEAM 2 or the irrelevant murine IgG3 mAb 14C7 were prepared following the manufacturer’s protocol. As F1, F2, and F3 were the only fractions to react in an immuno-dot blot (see, for example, Additional file 2, Supplementary Fig. S1), they were the only fractions that were further purified through co-immunoprecipitation. Each fraction was incubated separately with SEAM 2- or 14C7-linked magnetic beads. The beads were separated using a magnet, washed with the respective extraction buffer alone, and then with buffer containing polySia (50 μg/mL; colominic acid from Sigma-Aldrich) to remove nonspecific binding antigens. Finally, buffer containing 50 μg/mL N-propionyl polysialic acid [22] with 36% de-N-acetyl polysialic acid (N-Pr dPSA) was used to elute the antigens binding specifically to SEAM 2 from each fraction.

### Western blot of anti-nucleolin mAb MS-3 co-immunoprecipitated proteins

The anti-nucleolin mAb MS-3 linked to agarose beads (Santa Cruz Biotechnology) was used to co-immunoprecipitate nucleolin from the membrane fraction (F2) prepared from CHP-134 and SK-MEL-28 cells. After washing the MS-3-agarose beads two times with ProteoExtract® Subcellular Proteome Extraction buffer II, the bound proteins were eluted from the beads with ProteoExtract® buffer IV. Proteins eluted from the MS-3-agarose beads were resolved on 4–12% SDS-PAGE (NuPAGE, Thermo Fisher Scientific) and either stained with SimplyBlue® Coomasie stain (ThermoFisher) or transferred to a PVDF membrane (Immobilon®-FL, Millipore) using a semi-dry transfer cell (Trans-Blot® SD, Bio-Rad, Hercules, CA) for Western blotting. The PVDF membrane was blocked overnight with 5% dry whole milk in phosphate buffered saline (PBS) buffer then each pair of CHP-134 and SK-MEL-28 immunoprecipitates was stained with anti-dPSA mAb SEAM 3 (5 μg/mL) or MS-3 (1 μg/mL) or irrelevant mouse IgG (5 μg/mL) in blocking buffer for 2 h at ambient temperature. After washing three times with PBS buffer the bound antibodies were detected with IRDye® 800CW-conjugated donkey anti-mouse IgG (H + L) secondary antibody (LI-COR, Lincoln, NE). Images of gels and blots were recorded on an Odyssey® Fc Imaging System (LI-COR).

### dPSA ELISA

dPSA was produced by heating a solution (10 mL) of 100 mg of colominic acid (MilliporeSigma) and 10 mg sodium cyanoborohydride (MilliporeSigma) in 2 M NaOH to 100 °C in a sealed hydrolysis tube (ThermoFisher Scientific) for 40 min. The solution was cooled, neutralized with 2 M HCl, dialyzed (Spectra/Por™, Fisher Scientific) two times with 4 L of water and lyophilized. dPSA containing 35% de-N-acetyl neuraminic acid residues, as determined by a modified resorcinol assay [30], was conjugated to biotin as described previously [28]. The ELISA was performed with serial dilutions of nucleolin or SEAM 3 as in the absence or presence of 50 μg/mL of colominic acid or unmodified dPSA as described previously [28].

### LC/MS/MS protein identification

The eluted proteins from each cell fraction were resolved on 4–12% gradient SDS-PAGE gels (Thermo Fisher Scientific). The gels were stained with SimplyBlue™ Coomassie stain (Thermo Fisher Scientific). The excised gel sections were extracted overnight in 50 mM ammonium bicarbonate containing 50% acetonitrile. The disulfide bonds were reduced and modified with iodoacetamide per University of California San Francisco In-Gel Digestion Protocol [31] prior to trypsin digest (Promega Corp., Madison, WI). LC/MS/MS protein identification of tryptic peptides was performed by the University of California Davis Proteomics Core facility. Peptides from sections from the irrelevant antibody control were combined for LC/MS/MS analysis.

### Laser scanning confocal microscopy

SK-MEL-28, *ST8SIA2* knockdown mutant, and scrambled RNA negative control cells (∼10^5^ cells/mL) were cultured on glass coverslips coated with human placental Type IV collagen (Sigma-Aldrich). After an overnight incubation, cells were fixed with 4% formaldehyde for 1 h in PBS. For staining internal antigens, coverslips were treated with ice-cold 0.25% Triton X-100 in PBS for 10 min. The coverslips were blocked in blocking buffer (2% goat serum in PBS/0.25% Tween). Primary antibodies (5 μg/mL) in blocking buffer were added to the coverslips and incubated at ambient temperature. After PBS/Tween washes, goat anti-mouse isotype-specific secondary antibodies conjugated to Alexa Fluor 488 or Alexa Fluor 594 (Thermo Fisher Scientific) were added (1:200 dilution) in blocking buffer at room temperature in the dark. Subsequently, the cells were washed with PBS/Tween, then PBS. Finally, DNA was stained with 4′,6-diamidino-2-phenylindole (DAPI) in PBS for 10 min before mounting with mounting medium (Electron Microscopy Sciences, Hatfield, PA). Confocal images were obtained using a Zeiss LSM710 laser scanning confocal microscope and analyzed using ImageJ Software [32] and JACoP [33].

### Constructing knockdown cell lines targeting *ST8SIA2* and *ST8SIA4* by RNA interference

Knockdown cell lines were produced in SK-MEL-28 human melanoma cells by vector-based interfering RNA using a BLOCK-iT™ Pol II miRNA RNA vector system (Thermo Fisher Scientific). SK-MEL-28 cells were transfected with one of four vector constructs, two targeting each gene, called pcDNA 6.2-GW/+ EmGFP – PolyST, where PolyST represents ST8SIA2–1, ST8SIA2–2, ST8SIA4–1, and ST8SIA4–2. An additional scrambled control construct which does not target any known vertebrate gene was made, for a total of five vector constructs.

For each construct, multiple clonal cells lines were established. Following the Thermo Fisher Scientific TurboFect Transfection Reagent general protocol (up to step 7), approximately 5 × 10^4^ cells were seeded in each 24-well plates with growth medium 24 h prior to transfection. Cells were trypsinized and replated after transfection into 6-well plates (continuing with step 3 of the BLOCK-iT™ Pol II miR RNAi Expression Vector Kits - Generating Stable Cell Line protocol), then selected for survival in medium with 5 μg/mL blasticidin. Blasticidin-resistant cell lines were cloned by limiting dilution and a subset of 4 scrambled control constructs, 7 ST8SIA2–1 constructs, 2 ST8SIA2–2 constructs, 4 ST8SIA4–1 constructs, and 3 ST8SIA4–2 constructs were screened by fluorescence microscopy. Cells with EmGFP fluorescence were identified as cells containing the integrated plasmid.

Integration of the vector sequence was confirmed by isolating the genomic DNA (5 Prime PerfectPure DNA Cultured Cell Kit, Thermo Fisher Scientific) from 20 cell lines, amplifying the DNA target sequence with conventional PCR (MJ Research, Inc., South San Francisco, CA) using a T7 primer and running the products on a 0.6% agarose gel containing 0.01% ethidium bromide. After cutting out the PCR products from the gel (Qiagen QiaQuick Gel Extraction kit, Thermo Fisher Scientific), the PCR product was sequenced (Sequetech, Mountain View, CA).

### Quantifying expression of polysialyltransferase mRNA using real-time qPCR

RNA from the cells was isolated (Fermentas GeneJet RNA purification kit, Thermo Fisher Scientific), and cDNA was produced (Fermentas Maxima First Strand cDNA Synthesis Kit) for use in a Taqman Gene Expression real-time qPCR assay (Thermo Fisher Scientific). The relative mRNA copy numbers of *ST8SIA2* or *ST8SIA4* was determined using glyceraldehyde-3-phosphate dehydrogenase (GAPDH) as an internal control for each of the clonal cell lines (in triplicate samples). Cell lines with decreased mRNA copy number compared to a control cell line with integrated plasmid and cultured in the presence of blasticidin were propagated for further study.

### CRISPR knockout of ST8SIA2 and ST8SIA4 genes in CHP-134 cells

CHP-134 cell lines with *ST8SIA2* and *ST8SIA4* knocked out individually and in combination were constructed by WuXi AppTec Co. Ltd. (Shanghai, China) The sequences ataaccagacgctctctctg and actatgtgcttgacaggcgc were targeted for knocking out *ST8SIA2* and *ST8SIA4*, respectively. After confirming biallelic gene knockouts by reverse transcribing mRNA to cDNA and cloning and sequencing the cDNA, the cell lines were further subcloned a second time as described above for SK-MEL-28 RNAi mutants to ensure clonality.

### Antibody binding to cells

FACS binding experiments were performed with CHP-134 wild-type and CRISPR knockout clone cells as described previously [22]. Cells were suspended in fresh RPMI 10% FBS medium. The cell count was adjusted to 10^6^ live cells/mL. Test Abs and controls were added (10 μg/mL) to cells in tubes and incubated at ambient temperature for 45 min while mixing by mechanically rotating the tubes. The cells were washed with RPMI 10% FBS, then resuspended in the same medium containing secondary goat anti-mouse IgG AlexFluor 647 (Thermo Fisher Scientific). After 30 min incubation with mixing, the cells were washed once with Dulbecco’s phosphate buffered saline (DPBS) without Mg^2+^ or Ca^2+^ salts and suspended in DPBS containing 0.5% (volume/volume) formaldehyde for 10 min at room temperature. Binding was analyzed using a LSR Fortessa Flow Cytometer (BD Biosciences, San Jose, CA). FlowJo (TreeStar, Woodburn, OR) was used for data analysis.

## Results

### Anti-dPSA and anti-nucleolin co-immunoprecipitate dPSA and nucleolin from human cancer cells

**Fig. 1:**
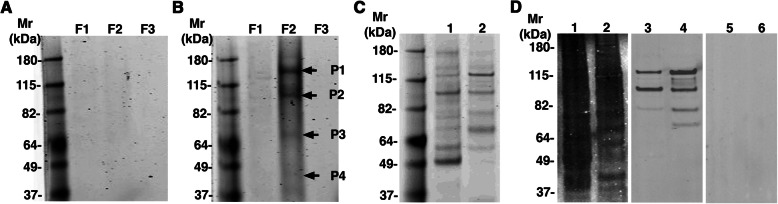
Anti-dPSA and anti-nucleolin mAbs co-immunoprecipitates nucleolin and dPSA from the SK-MEL-28 and CHP-134 membrane fractions. Differential detergent extraction was used to separate human melanoma SK-MEL-28 cells into subcellular fractions for co-immunoprecipitation with a control murine IgG3 mAb (14C7) or anti-dPSA mAb SEAM 2 (both are subtype IgG3) linked to magnetic beads. Fractions F1, F2, and F3 are cytoplasmic, cell membrane, and nuclear fractions, respectively. Also, anti-nucleolin mAb MS-3 linked to agarose beads was used to co-immunoprecipitate proteins from the membrane fraction prepared from CHP-134 and SK-MEL-28 cells. Sections of the SimplyBlue® Coomassie-stained gels in lanes labeled F2 indicated by arrows and labeled P1–P4 of both gels were excised and proteins contained in them identified by in-gel trypsin digestion and LC/MS/MS peptide mass fingerprinting. **A** Proteins co-immunoprecipitated by the irrelevant IgG3 mAb. **B** Proteins co-immunoprecipitated by SEAM 2. **C** SDS-PAGE gel of proteins co-immunoprecipitated from the membrane fraction of CHP-134 (lane 1) and SK-MEL-28 (lane 2) with MS-3. (**D**) Western blots of MS-3-co-immunoprecipitated proteins shown in (**C**) stained with anti-dPSA mAb SEAM 3 (lanes 1 and 2), MS-3 (lanes 3 and 4), and irrelevant mouse IgG (lanes 5 and 6)

To determine the identity of antigens potentially modified with dPSA, we prepared cytoplasmic, cell membrane, nuclear, and cytoskeletal fractions (F1–F4, respectively) from human SK-MEL-28 melanoma cells. The fractions were combined with anti-dPSA mAb SEAM 2 or an irrelevant control murine IgG3 mAb (14C7) covalently linked to magnetic beads. The cytoskeletal fraction was not subjected to co-immunoprecipitation since this fraction, which contains SDS, was not reactive with SEAM 2 in an immunodot blot (Additional file 2, Supplementary Fig. S1). The proteins bound to the beads were specifically eluted with N-Pr polySia containing 36% de-N-acetyl sialic acid in extraction buffer after a wash with buffer containing polySia. SEAM 2 has more than 100 times greater avidity for the N-Pr polySia dPSA antigen than dPSA containing N-acetyl sialic acid residues based on an inhibition ELISA [19]. The eluted proteins were resolved on SDS-PAGE gels. As shown in Fig. 1, the sample from the SEAM 2 co-immunoprecipitated membrane fraction (Fig. 1b, lane F2) contained the majority of Coomassie-stained proteins. The proteins appear largely as a “smear” rather than distinct bands. Typically, proteins modified with polySia run in SDS-PAGE gels over a relatively wide range of apparent mass because of variable polySia length. Variable amounts of polySia de-N-acetylation may result in additional heterogeneity. Four relatively dark-staining sections of the gel spanning the range of eluted dPSA antigens were excised from the gel and processed to generate tryptic peptides for LC/MS/MS mass fingerprinting (indicated by arrows in Fig. 1b, lane F2). As controls, gel sections were taken from the same relative positions in the sample from the membrane fraction control irrelevant IgG3 mAb co-immunoprecipitation and were processed similarly but the tryptic peptides from all fractions were combined in one sample for LC/MS/MS analysis (Additional file 1) since only trace amounts of peptides were obtained (Fig. 1a, lane F2).

The tryptic peptide LC/MS/MS analysis of the bands excised from the SEAM 2-F2 immuno-precipitate identified nucleolin (14 exclusive peptides, 16 exclusive unique spectra, 167/710 amino acids identified in the segment including residues 139–624) as the predominant protein in slices marked P1 (~ 145 kDa) and P2 (~ 115 kDa). In addition, there were trace amounts of several ribosome-associated proteins in both fractions. One function of nucleolin is to promote assembly of ribosomes in nucleoli. Slices marked P3 (~ 71 kDa) contained ribosome-associated proteins and α-tubulin in addition to nucleolin, and P4 (~ 48 kDa) contained ribosome-associated proteins and actin. There were no cell-derived proteins identified in the combined control sample (Additional file 1). The calculated molecular mass of nucleolin is 77 kDa, but nucleolin can have several apparent masses ranging from ~ 50 kDa to ~ 100 kDa because of multiple covalent modifications including sialylation, the presence of highly acidic regions near the N-terminal region, and self-cleaving activity [34, 35]. The experiment was repeated and included an additional control of co-immunoprecipitation with the anti-polySia mAb SEAM 12 (Additional file 2, Supplementary Fig. S2). Again, nucleolin was the predominant protein co-immunoprecipitated by SEAM 2 from the membrane fraction in gel slices corresponding to lane F2 bands P1 and P2 in Fig. 1b (38 exclusive unique peptides, 53 exclusive unique spectra, 274/710 amino acids identified in the segment including residues 73–629), with smaller amounts of nucleolin also present in the SEAM 12 co-immuno-precipitate, and trace amounts in the irrelevant control. To determine whether dPSA was unique to SK-MEL-28 cells or common to all dPSA containing cells, the same co-immunoprecipitation procedure of subcellular-enriched cell fraction F2 was repeated for Kelly neuroblastoma, SNU-1 gastric cancer cells, and normal PBMCs (Additional file 2, Supplementary Figs. S3 and S4, respectively). All displayed anti-dPSA reactivity inside and outside cells by FACS and fluorescence microscopy except for PBMCs, which had anti-dPSA reactivity with both SEAM 2 and SEAM 3 inside cells (i.e., detergent-treated) but not on the surface of cells not treated with detergent to permeabilize them (Additional file 3, Supplementary Fig. S7). The cells tested represent a range of *ST8SIA2* and *ST8SIA4* expression based on RNA-seq data from publicly available databases [36]: Kelly having high *ST8SIA2* and low *ST8SIA4*; PBMCs having no *ST8SIA2* and high *ST8SIA4*; and SNU-1 having low *ST8SIA2* and *ST8SIA4*. Nucleolin was co-immunoprecipitated by SEAM 2 from the membrane fraction (F2) of Kelly (19 exclusive unique peptides, 22 exclusive unique spectra, 190/710 amino acids identified in the segment including residues 73–649), SNU-1 (20 exclusive unique peptides, 32 exclusive unique spectra, 314/710 amino acids identified in the segment including residues 73–648), and PBMCs (10 exclusive unique peptides, 14 exclusive unique spectra, 214/710 amino acids identified in the segment including residues 80–648) that was not present in corresponding gel slices of the irrelevant negative control. Importantly, there were no peptides observed in any of the anti-dPSA immunoprecipitation experiments identifying proteins known to be modified with polySia [37]. Therefore, none of the known polySia-modified proteins appear to be modified with dPSA.

Next, we co-immunoprecipitated proteins from the membrane fraction (F2) prepared from CHP-134 and SK-MEL-28 cells using anti-nucleolin mAb MS-3 linked to agarose beads. The proteins eluted from the beads were resolved by SDS-PAGE, stained with Simply Blue®, and reactivity with anti-dPSA mAb SEAM 3, MS-3 and irrelevant mouse IgG on Western blots is shown in Fig. 1, panels C and D, respectively. A set of relatively high molecular mass proteins (> 37 kDa- < 180 kDa), like those observed for co-immunoprecipitation with SEAM 2, was obtained by co-immunoprecipitation with MS-3. The proteins co-immunoprecipitated with MS-3 were strongly reactive with SEAM 3, confirming that dPSA is directly linked to nucleolin.

The number of MS-3-reactive proteins on the Western blot from the membrane fraction of CHP-134 cells was less than the number from SK-MEL28 cells (Fig. 1d, lane 4 versus lane 3) and the faster migrating dPSA antigens were not reactive with MS-3 at all (Fig. 1d, lanes 1 and 2 versus lanes 3 and 4). Since CHP-134 cells express considerably more dPSA than SK-MEL-28 cells, the result suggests that dPSA may block the epitope recognized by MS-3. There are potentially many SEAM 3 epitopes since polySia can be hundreds of residues long while the SEAM 3 epitope is approximately 4 residues [22]. The larger number of potential SEAM 3 epitopes may account for the much greater, broader reactivity of SEAM 3 compared to MS-3.

Since nucleolin is not known to be polysialylated but does form complexes with RNA and DNA, it is possible that nucleolin associates with dPSA non-specifically resulting in co-immunoprecipitation by anti-dPSA. To address this possibility, we performed an ELISA assay with dPSA absorbed to the plate. The starting concentration of purified nucleolin used in the assay was based on assuming 1 million molecules of nucleolin on the cell surface, 10 million cells extracted in a final volume of 1 mL and 0.25 mL used for immunoprecipitation (~ 10 nM). As shown in Additional file 2, Supplementary Figure S5, no nucleolin binding to dPSA above background was detected either in the absence or presence of polySia (colominic acid) or dPSA. The EC_50_ for SEAM 3 binding in the same assay was 0.27 nM and binding was inhibited by soluble dPSA.

### Anti-dPSA is co-localized with nucleolin on the surface of SK-MEL-28 melanoma cells

**Fig. 2:**
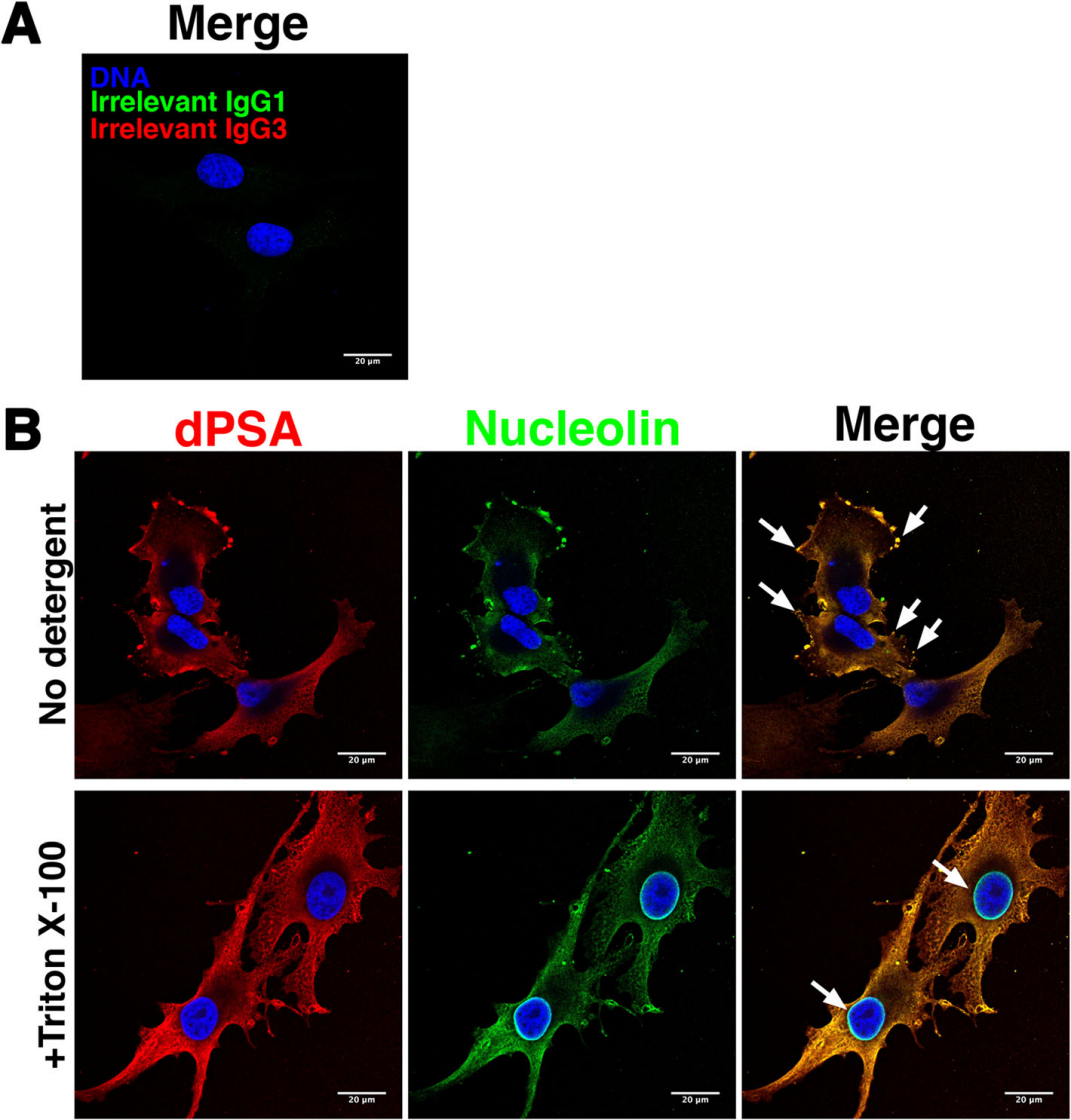
dPSA is co-localized with nucleolin on the cell surface and inside SK-MEL-28 human melanoma cells. dPSA and nucleolin were identified on the surface (no detergent) or inside (+Triton X-100) SK-MEL-28 cells with anti-dPSA mAb SEAM 2 or anti-nucleolin mAb MS-3, respectively, and detected by fluorescently labeling with Alexa Fluor 594 (red fluorescence) or Alexa Fluor 488 (green fluorescence)-conjugated anti-mouse subtype-specific antibodies using confocal laser scanning microscopy. Blue fluorescence is DAPI DNA staining. **A** Cells with irrelevant murine IgG1 and IgG3 and secondary antibodies and (**B**) cells with SEAM 2 and MS-3; the arrows in merge image indicate dPSA concentrated in lamellipodia, podosomes, and filopodia in “No detergent” micrographs. Arrows in merge image of +Triton X-100-treated cells show nuclear nucleolin, which is not marked by mAb SEAM 2. Scale bars, 20 μm

Nucleolin is a nuclear protein, but it is also found on cilia of airway epithelial cells, where it serves as a receptor for various pathogens [38] and has been reported to be present on the cell surface of cancer cells [39]. Since anti-dPSA mAb SEAM 2 co-immunoprecipitated nucleolin, we asked whether nucleolin is co-localized with dPSA in SK-MEL-28 cells. Anti-nucleolin mAb MS-3 and anti-dPSA mAb SEAM 2 were used to detect each antigen combined with subclass-specific fluorescently labeled secondary antibodies by laser scanning confocal fluorescence microscopy. As a control, the cells were treated with an irrelevant murine IgG3 mAb and the secondary antibodies. As shown in Fig. 2a, there was no staining with irrelevant mAbs or secondary antibodies alone. In wild-type SK-MEL-28 cells, dPSA (Fig. 2b, red fluorescence) was present on the surface of cells and was concentrated in lamellipodia, podosomes, and filopodia (examples indicated by arrows in Fig. 2b, merge image). Anti-nucleolin staining (Fig. 2b, green fluorescence) was identical with respect to location and intensity of staining to anti-dPSA staining (Fig. 2b, merge).

### Membrane-associated dPSA-nucleolin is distinct from nuclear nucleolin

Next, we looked at staining patterns inside cells by treating fixed SK-MEL-28 cells with the detergent Triton X-100 to permeabilize them and allow entry of primary and secondary antibodies. dPSA inside cells exhibited a web-like pattern typical of smooth endoplasmic reticulum and trans golgi network (Fig. 2b, +Triton X-100). Again, anti-nucleolin staining matched that of anti-dPSA inside cells with respect to location and intensity of staining. The exception was the strong anti-nucleolin staining around the nuclear membrane, which was not reactive with anti-dPSA mAbs (Fig. 2b, +Triton X-100).

### Effect on dPSA and cell surface nucleolin of knocking down expression of *ST8SIA2* with siRNA in human SK-MEL-28 cells

**Fig. 3:**
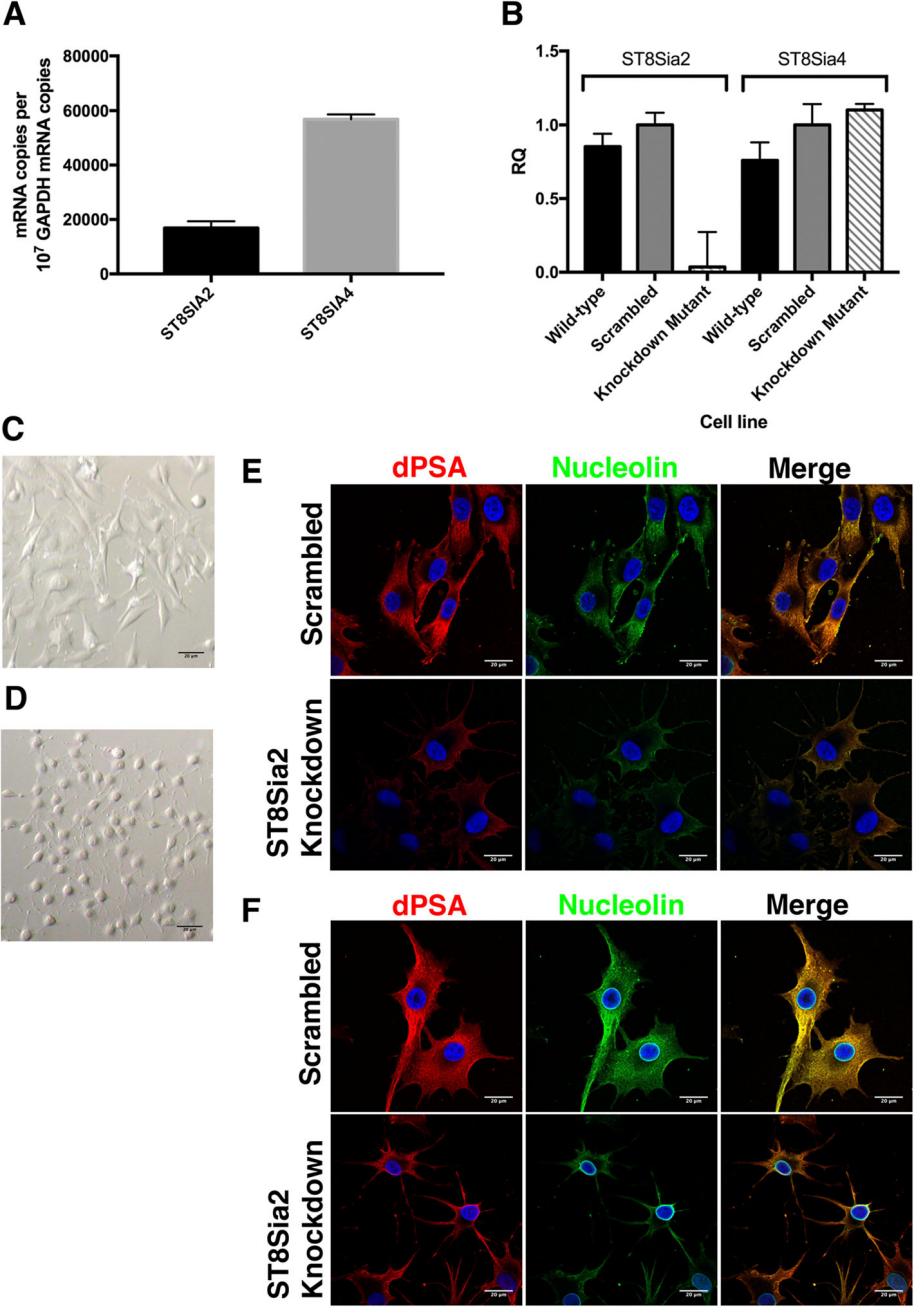
RNAi knockdown of ST8SIA2 decreases expression of membrane-associated dPSA and nucleolin but not nuclear nucleolin. RNAi was used to knockdown expression of ST8SIA2 in SK-MEL-28 human melanoma cells and the effect on dPSA and nucleolin production was determined by confocal laser scanning microscopy. **A** The absolute *ST8SIA2* and *ST8SIA4* mRNA copy number relative to that of GAPDH in parent SK-MEL-28 cells. *N* = 3. **B** Relative expression of mRNA coding for ST8SIA2 and ST8SIA4 polysialyltransferases in parent, a scrambled RNA negative control and *ST8SIA2* RNAi knockdown SK-MEL-28 mutants measured by RT-PCR. N = 3. Brightfield micrographs of (**C**) scrambled control compared to (**D**) *ST8SIA2* knockdown mutant show morphological differences. Confocal laser scanning micrographs of the scrambled and *ST8SIA2* knockdown mutant show dPSA and nucleolin are co-localized on (**E**) the surface and (**F**) inside cells but are both decreased when *ST8SIA2* is knocked down except for nucleolin in the nuclear membrane. dPSA and nucleolin were identified on the surface (**E–F**, upper panels) or inside (treated with Triton X-100; **E–F**, lower panels) SK-MEl-28 cells with anti-dPSA mAb SEAM 2 or anti-nucleolin mAb MS-3, respectively, and detected by fluorescently labeling with Alexa Fluor 594 (red fluorescence) or Alexa Fluor 488 (green fluorescence)-conjugated anti-mouse subtype-specific antibodies using confocal laser scanning microscopy. Blue fluorescence is DAPI DNA staining. Scale bars, 20 μm

SK-MEL-28 cells express both *ST8SIA2* and *ST8SIA4* (Fig. 3a). Previously, we showed that transient *ST8SIA4* RNAi knockdown in SK-MEL-28 cells decreased production of polySia and dPSA, demonstrating that dPSA was derived from polySia [22]. In this study, we constructed mutant SK-MEL-28 cell lines by chromosomal integration of plasmids expressing siRNA targeting *ST8SIA2*. The relative expression of both genes in total mRNA was determined by quantitative real-time PCR and normalized using GAPDH mRNA as in internal control (Fig. 3a). There was a significant reduction in the amount of *ST8SIA2* mRNA in the *ST8SIA2* knockdown compared to the wild-type and the scrambled control constructs (Fig. 3b). There was no effect in the *ST8SIA2* knockdown mutant on expression of *ST8SIA4* mRNA (Fig. 3b), which was ~ 3-fold higher than the wild-type level of *ST8SIA2* mRNA (Fig. 3a).

RNAi knockdown of *ST8SIA2* resulted in altered cell morphology (Fig. 3d) compared to the negative control cell line expressing a scrambled RNA (Fig. 3c). The cells with *ST8SIA2* RNAi knockdown had a distinct rounded appearance with relatively short lamellipodia circularly arrayed around the cell (Fig. 3d).

Cell surface dPSA and nucleolin were decreased in the *ST8SIA2* RNAi knockdown mutant compared to the control SK-MEL-28 mutant expressing a scrambled RNA (Fig. 3e). Similarly, the cytoplasmic amounts of dPSA and nucleolin in Triton X-100-treated cells (Fig. 3f) were reduced in the *ST8SIA2* knockdown mutant. siRNA had no effect on anti-nucleolin staining of the nuclear membrane (Fig. 3f) showing dPSA-modified and nuclear nucleolin were distinct forms of the protein.

### CRISPR knockout of *ST8SIA2* but not *ST8SIA4* eliminated cell surface dPSA in human CHP-134 neuroblastoma cells

**Fig. 4:**
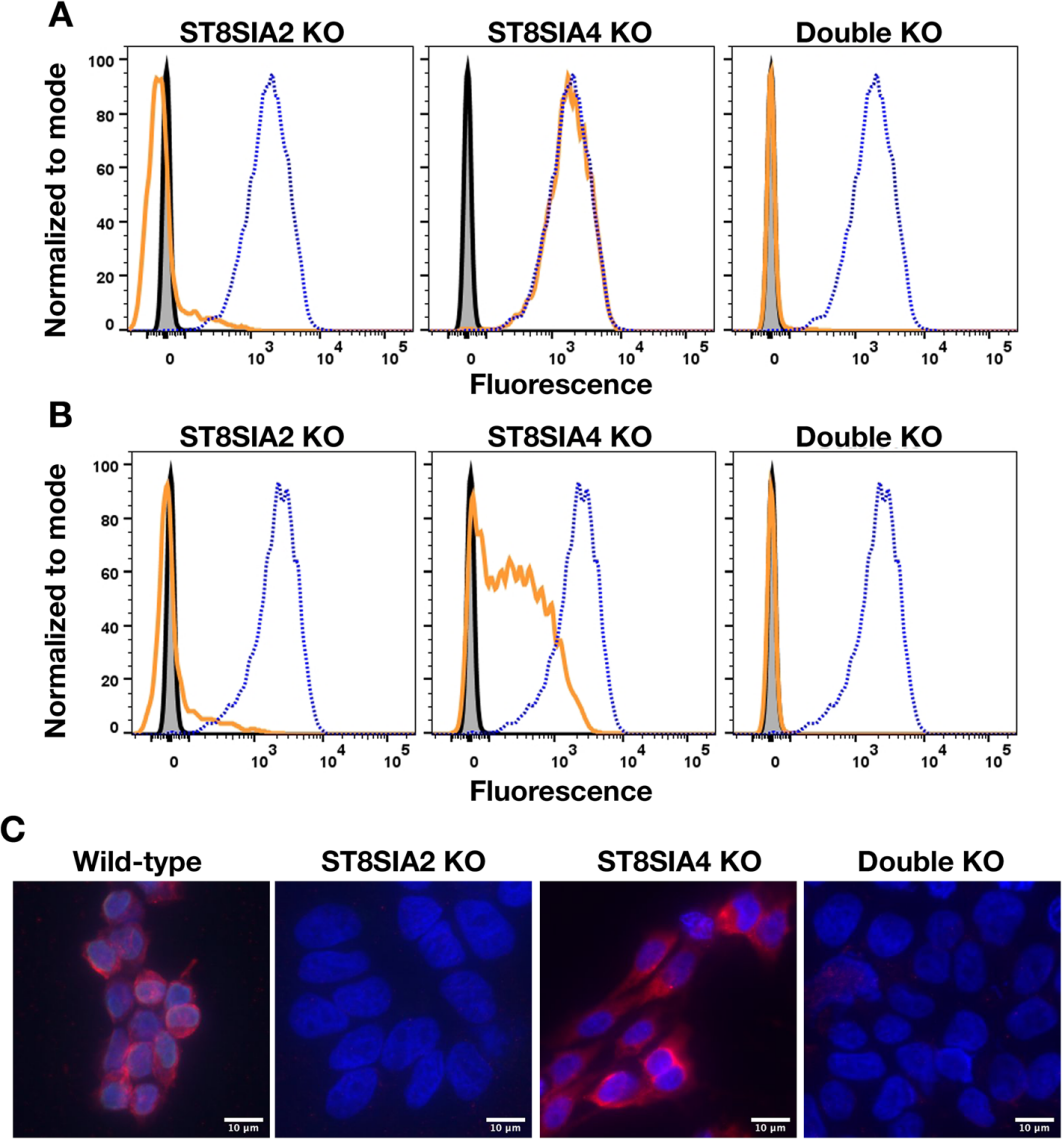
Knocking out (KO) *ST8SIA2* eliminates cell surface dPSA and PSA from CHP-134 cells. Cell surface dPSA (**A**) and polySia (**B**) on live wild-type (dotted blue line) and the indicated mutants (solid orange lines) detected by FACS using anti-dPSA mAb SEAM 3 and anti-polySia mAb SEAM 12 as primary antibodies. Controls included no primary antibody (gray-filled histograms) and irrelevant IgG2b or IgG2a mAbs, respectively (solid black lines). Fluorescence micrographs (**C**) of wild-type CHP-134 transfected with an empty plasmid compared to the CRISPR knockout cell lines of the indicated genes. Red fluorescence is anti-dPSA (SEAM 3); blue fluorescence is DAPI DNA staining. Scale bars, 10 μm

To determine whether cell surface dPSA depends on the activity of one or both polysialyltransferases, we used CRISPR to knock out each gene individually and in combination in human neuroblastoma CHP-134 cells. CHP-134 cells were chosen since both genes are relatively highly expressed [22] and we wanted to determine whether the effects of interfering with expression of the polysialyltransferases in SK-MEL-28 cells could be replicated in another cancer cell line by a different method. Cell lines transfected with plasmids coding for Cas9 and RNAs targeting the respective polysialyltransferase gene(s) were cloned, and the targeted gene knockouts were confirmed. When *ST8SIA2* alone or in combination with *ST8SIA4* was knocked out, cell surface dPSA, as measured by FACS and fluorescence microscopy with SEAM 3, was eliminated (Fig. 4a). Interestingly, knocking out *ST8SIA2* also decreased cell surface polySia (Fig. 4b) suggesting that, in CHP-134 cells, polysialyltransferases may work synergistically as suggested by Angata et al. [17]. PolySia in CHP-134 cells mainly modifies NCAM [40]. Knocking out *ST8SIA4* had no effect on cell surface dPSA but reduced cell surface polySia (Fig. 4a and b, respectively). Therefore, cell surface dPSA in CHP-134 cells depended entirely on expression of *ST8SIA2*.

Unlike SK-MEL-28 cells, there were no apparent differences in the morphology of CHP-134 mutants. However, nuclei in cell lines with *ST8SIA2* and *ST8SIA2 + ST8SIA4* knocked out were larger (on average 1.45 ± 0.24 times greater based on length times width measurements of 20 nuclei each) compared to wild-type or the *ST8SIA4* knockout cell lines (Fig. 4c). The differences in the size of the nuclei depended on cell attachment to a surface, as there were no differences in the size of the nuclei of cells in suspension.

Intracellular antigens reactive with both SEAM 2 and SEAM 3 were reduced but still detected in detergent-treated cells after knocking out *ST8SIA2*, *ST8SIA4* or both genes in CHP-134 cells and in normal PBMCs that do not express *ST8SIA2*. Immunodot blots of cell fractions from wild-type CHP-134 cells compared to *ST8SIA2* and *ST8SIA2 + ST8SIA4* knockout cell lines show that anti-dPSA Ab reactivity with the cytoplasmic fraction is not dependent on *ST8SIA2* or *ST8SIA4* (Additional file 2, Supplementary Fig. S6). The results suggest that most of the internal staining described previously [21] may result from reactivity with a de-N-acetyl sialic acid antigen produced by another sialyltransferase [26, 41, 42]. However, the protein identified by immunoprecipitation and mass spectroscopy in both cancer cells and normal PBMCs was nucleolin. Taken together, the data suggest nucleolin modified with de-N-acetyl sialic acid may be an anchor glycan that, when polysialylated by ST8SIA2 and de-N-acetylated further, results in transfer of intracellular dPSA-nucleolin to the cell surface.

### dPSA-nucleolin is present on the surface of primary and metastatic pancreatic, gastric, and ovarian cancer cell lines

To determine whether dPSA co-localization with nucleolin was unique to SK-MEL-28 cells or common to cancer cells, we performed similar confocal fluorescence microscopy experiments labeling dPSA with SEAM 2 and nucleolin with anti-nucleolin mAb MS-3 on sets of seven pancreatic, five gastric, four ovarian primary and metastatic cell lines (Additional file 3, Supplementary Figs. S8-S10, respectively). The collections of cell lines represent different phenotypic and genetic characteristics for each type of cancer. The panels are based on key components of cell signaling pathways or cancer genes. The cell lines within the molecular signature panels were obtained directly from and have been fully validated by ATCC to verify mutations, gene copy number changes, gene expression, protein expression and cell functionalities. Control antibodies included irrelevant murine IgG1 and IgG3 mAbs and Alexa Fluor-conjugated secondary antibodies (Additional file 3, Supplementary Figs. S8-S10). Except for the ovarian teratocarcinoma cell line PA-1 (Additional file 3, Supplementary Fig. S9), all the cancer cell lines were positive for both dPSA and cell surface nucleolin, which were co-localized as determined by analysis of Z-stack confocal micrographs using JACoP [33]. dPSA staining was much stronger and consistent for all cell lines that were positive for dPSA, whereas anti-nucleolin staining was less consistent with anti-nucleolin being less reactive for cell lines having high polysialyltransferase expression. There was no surface anti-dPSA or nucleolin staining on human normal PBMCs (Additional file 3, Supplementary Fig. S7), which include subsets of cells with high levels of *ST8SIA4* and polySia-NCAM expression.

### ST8SIA2 is abnormally expressed in many cancers

**Fig. 5:**
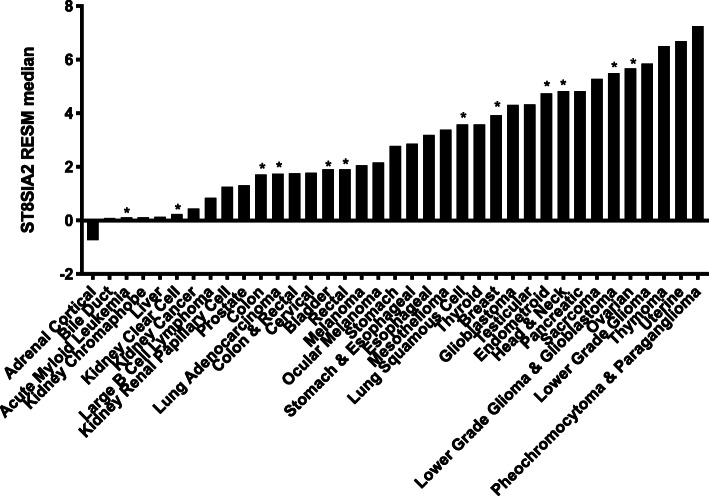
ST8SIA2 is re-expressed in most of the 37 human cancers included in TCGA. mRNA expression from RNA-seq data is shown relative to the Pan-Cancer [43] median (Pan-Cancer median expression is determined from cancers indicated by asterisks over the bars)

Based on recent data from publicly available RNA-seq and proteomics databases, *ST8SIA2* expression in cells of adult normal tissues is either very low or non-existent (see, for example, GeneCards: the human gene database [3]). However, based on RNA-seq data from The Cancer Genome Atlas (TCGA) Program [36] for 37 cancers, most express some level of *ST8SIA2* that does not occur in corresponding cells of human normal tissues (Fig. 5). The *ST8SIA2* gene expression data are consistent with the reported prevalence of dPSA-linked cell surface nucleolin in human cancers [44]. Based on combined gene expression and proteomics data, the polysialyltransferase does not appear to be produced at measurable levels in any adult normal tissue. However, as shown in Fig. 5, higher expression is observed in most cancers where it is associated with invasiveness, metastasis, and poor prognosis [45].

## Discussion

Altered glycosylation patterns of cell surface proteins occur in nearly all types of cancer. Excessive sialylation of glycoproteins and glycolipids is central to the aberrant regulation of cell adhesion in metastatic cancer, which in turn can result from re-expression and/or overexpression of genes normally expressed during development but not in cells of adult normal tissues. In this study, we gathered further evidence that dPSA is a novel tumor-associated carbohydrate antigen. We have shown that dPSA is linked to or associated with nucleolin, which is normally a nuclear protein but is reported to be on the cell surface of many cancers [44]. We have also shown that the presence of dPSA-nucleolin on the surface of cancer cells appears to be dependent on re-expression of a fetal polysialyltransferase gene, *ST8SIA2*, which may indicate a novel cancer pathway to further investigate for additional points of therapeutic intervention.

Protein polysialylation is highly restricted. To date, only six proteins in humans are reported to be modified with polySia: NCAM [5], E-selectin ligand-1 [7], the scavenger receptor CD36 [9], neuropilin-2 [4], SynCAM1 [46], and ST8SIA2 itself [6]. In this study, none of the previously known polysialylated proteins in human cells were co-immunoprecipitated from any of the cancer cell lines tested or human normal PBMCs with anti-dPSA antibodies, suggesting that dPSA modification or association is exclusive to nucleolin. While nucleolin does not appear to bind directly to dPSA in ELISA (Additional file 2, Supplementary Figure 5S), there remains the possibility that nucleolin may associate with an unidentified molecule that is modified with dPSA. The large size and electrostatic repulsion of polyanionic polySia is thought to block homophilic interactions between adhesion molecules such as NCAM [10] during nervous system development. However, partial de-N-acetylation, which is common to many polysaccharides [27], alters the charge of polySia as de-N-acetylation produces zwitterionic polysaccharides (ZPS) with different chemical and biological properties. For example, microbial ZPS are frequently involved in immune shielding such as formation of biofilms [47], modulating T cell function [48], and have increased immunogenicity compared to N-acetylated polysaccharides [30].

Nucleolin is an RNA binding protein found throughout the cell where it has been detected in the cytoplasm, nucleus, nucleoli, cell membrane and on cilia [35]. The reason for the difference in nucleolin subcellular localization is thought to be the result of post-translational modifications, which include sialylation—although polysialylation of nucleolin was not known to occur previously [49]. However, in cancerous cells but not normal cells, nucleolin is reported to be expressed consistently and abundantly on the cell surface [44]. Cell signaling pathways including transforming growth factor-β, epidermal growth factor receptor and Fas receptor, which promote proliferation and survival of cancer cells, have been linked to cell surface nucleolin (reviewed in [50]). The apparently ubiquitous presence of cell surface nucleolin in cancer and its possible role in several hallmarks of cancer has simulated considerable interest in therapeutic approaches targeting nucleolin (reviewed in [50]).

Previously, we reported that internal staining in SK-MEL-28 cells was decreased after knocking down expression of *ST8SIA4* by transient transfection with siRNA targeting *ST8SIA4* [22]. That study focused on internal anti-dPSA reactivity since anti-dPSA reactivity with cell-surface dPSA was limited to a small fraction of cells and the mean fluorescence intensity observed by flow cytometry was close to background. Subsequently, we observed that adherent cell lines, such as SK-MEL-28 cells, treated with trypsin/EDTA to release them from the culture flask as done in that study, resulted in the removal cell surface dPSA. Presumably this occurred because of proteolysis of the nucleolin carrier. However, the *ST8SIA4* knockdown-dependent decrease in SEAM 3 reactivity with internal antigens was statistically significant in multiple experiments suggesting that ST8SIA4 may also have a role the production of internal, cytosolic de-N-acetylated polySia antigens. Unfortunately, the intracellular antigens reactive with anti-dPSA antibodies, which are prevalent in both normal and cancer cells [21] remain unidentified but appear to be unrelated to membrane-associated dPSA based on immune-dot blots of subcellular fractions from CHP-134 *ST8SIA2* and *ST8SIA2 + ST8SIA4* double knockout cell lines (Additional file 2, Supplementary Fig. S6). In contrast, cell surface dPSA depended on *ST8SIA2* expression. Knocking down or knocking out *ST8SIA2* in two different cell lines also reduced or eliminated cell surface nucleolin, respectively. In addition to the cancer cell lines, further evidence for the link between *ST8SIA2* and cell surface dPSA-nucleolin was shown with human normal PBMCs, which do not express *ST8SIA2* and do not have cell surface dPSA or nucleolin (Additional file 3, Supplementary Fig. S7). Although cumulative data from RNA-seq suggest a low level of *ST8SIA2* expression in human heart and brain, the presence of ST8SIA2 protein has not been confirmed and we did not find cell surface dPSA in either tissue during a immunohistochemical study of 18 major human organs [21]. Overall, the data presented here show that there are distinct functions of each polysialyltransferase with respect to the subcellular distribution of dPSA and its presence on nucleolin.

## Conclusions

Expression of genes coding for polysialyltransferases are restricted in adult human normal tissues but commonly expressed in most cancers. This study suggests that *ST8SIA2* expression and cell surface dPSA are limited to cancer cells and the developing embryo [1, 21]. Since anti-dPSA mAbs have direct cytotoxic effects on multiple cancer cell lines [22], approaches that target *ST8SIA2* and dPSA to prevent and treat cancers without adverse effect on normal cells may be possible. The presence of dPSA on human trophoblasts [21], microbial pathogens [23, 24, 30], and cancer cells [21, 22] raises the possibility that the function of dPSA may be related to an unrecognized mechanism of immune shielding, since all of the above have in common a need to avoid being targeted by immunological mechanisms of protection. Although approaches to target cell surface nucleolin in cancer have been developed [35, 50], dPSA and the dPSA biosynthetic pathway have potential advantages for the development of cancer diagnostics and therapeutics. As described here, cell surface dPSA appears to be produced only during fetal development and in human cancers [1, 14, 21, 22, 45]. dPSA epitopes are consistent across cancers [19, 20, 23, 30]. There are potentially a large number of dPSA epitopes per chain [40] that bring anti-dPSA Abs in close proximity for activating cells and complement. Finally, anti-dPSA Abs can directly induce apoptosis in cancer cells [22], and, if a role in immune shielding is confirmed, there is the potential to block that mechanism. Identifying nucleolin as the protein modified by or associated with dPSA and linking the production of cell surface dPSA to expression of *ST8SIA2* establishes new possibilities for investigating dPSA and its role in human biology and disease.

## Supplementary Information

**Additional file 1:.** LC/MS/MS Cluster Report Data File.

**Additional file 2: Supplementary Fig. S1.** Immunodot blot of SK-MEL-28 cell differential detergent extraction fractions. **Supplementary Fig. S2.** Antigens immunoprecipitated from SK-MEL-28 melanoma cells differential detergent extraction fraction 2. **Supplementary Fig. S3.** Antigens immunoprecipitated from Kelly neuroblastoma cells differential detergent extraction fraction 2. **Supplementary Fig. S4.** Antigens immunoprecipitated from PBMCs and SNU-1 gastric cancer cells differential detergent extraction fraction 2. **Supplementary Fig. S5.** dPSA ELISA with nucleolin and SEAM 3. **Supplementary Fig. S6.** Immunodot blot of CHP-134, *ST8SIA2* and *ST8SIA2*+*ST8SIA4* CRISPR knockout cell line differential detergent extraction fractions.

**Additional file 3: Supplementary Fig. S7.** Human peripheral blood mononuclear cells do not express cell-surface dPSA or nucleolin. **Supplementary Fig. S8.** Primary and metastatic gastric cancer cell lines express cell-surface dPSA and nucleolin. **Supplementary Fig. S9.** Primary and metastatic ovarian cancer cell lines express cell-surface dPSA and nucleolin. **Supplementary Fig. S10.** Primary and metastatic pancreatic cancer cell lines express cell-surface dPSA and nucleolin.

## Data Availability

The datasets supporting the conclusions of this article are included within the article and its additional files.

## Abbreviations

CRISPR: Clustered regularly interspaced short palindromic repeats
DPBS: Dulbecco’s phosphate buffered saline
dPSA: de-N-acetyl neuraminic acid-containing poly alpha2–8 N-acetyl neuraminic acid
N-Pr dPSA: N-propionyl polysialic acid with 36% de-N-acetyl polysialic acid
FACS: Fluorescence activated cell sorting
FBS: Fetal bovine serum
GAPDH: Glyceraldehyde-3-phosphate dehydrogenase
mAb: monoclonal antibody
NCAM: Neural cell adhesion molecule
PBMCs: Peripheral blood mononuclear cells
polySia: poly alpha2–8 N-acetyl neuraminic acid
RNAi: RNA interference
RNA-seq: mRNA sequencing
siRNA: small interfering RNA
ZPS: Zwitterionic polysaccharide
