# Supplementary material for: A cancer-unique glycan: de-N-acetyl polysialic acid (dPSA) linked to cell surface nucleolin depends on re-expression of the fetal polysialyltransferase ST8SIA2 gene
Source: J Exp Clin Cancer Res. 2021 Sep 20;40:293. doi: 10.1186/s13046-021-02099-y (PMC8451149; doi:10.1186/s13046-021-02099-y)
Supplement: Supplementary file 2 — Additional file 2: Supplementary Fig. S1. Immunodot blot of SK-MEL-28 cell differential detergent extraction fractions. Supplementary Fig. S2. Antigens immunoprecipitated from SK-MEL-28 melanoma cells differential detergent extraction fraction 2. Supplementary Fig. S3. Antigens immunoprecipitated from Kelly neuroblastoma cells differential detergent extraction fraction 2. Supplementary Fig. S4. Antigens immunoprecipitated from PBMCs and SNU-1 gastric cancer cells differential detergent extraction fraction 2. Supplementary Fig. S5. dPSA ELISA with nucleolin and SEAM 3. Supplementary Fig. S6. Immunodot blot of CHP-134, ST8SIA2 and ST8SIA2+ST8SIA4 CRISPR knockout cell line differential detergent extraction fractions. [file 13046_2021_2099_MOESM2_ESM.docx]

**Additional file 2: Supplementary Figs. S1-S5**

F1 F2 F3 F4 F1 F2 F3 F4


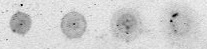

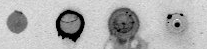


Irrelevant mAb 14C7 Anti-dPSA mAb SEAM 2

S**upplementary Fig. S1. Immunodot blot of SK-MEL-28 cell differential detergent extraction fractions**. Replicate portions of each detergent extract fraction from SK-MEL-28 cells prepared as described in Methods was spotted onto a PVDF membrane (Immobilon-FL, Millipore) mounted on a dot blot apparatus (Topac). After blocking with 2% non-fat dry milk in DPBS blocking buffer, the section of the membrane was incubated with the indicated mAb for 1 hr at ambient temperature in the same blocking buffer and washed with DPBS containing 0.1% Tween 20 three times for 5 minutes. mAbs binding to the membrane were detected with IRDye®800CW Donkey anti-Mouse IgG (H+L) secondary antibody (LI-COR) using the same method as the primary mAb and images were recorded on an Odyssey® Fc Imaging System (LI-COR).

1 2 3 4


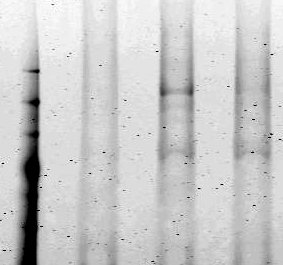


**Supplementary Fig. S2. Antigens immunoprecipitated from SK-MEL-28 melanoma cells differential detergent extraction fraction 2.** Immunoprecipitation was performed as described in the Methods using an irrelevant IgG3 mAb 14C7 (lane 2), anti-dPSA mAb SEAM 2 (lane 3) and anti-PSA mAb (SEAM 12). Proteins in the samples were resolved on 4–12% gradient SDS-PAGE gels (NuPAGE, Thermo Fisher Scientific). The gels were stained with SimplyBlue™ Coomassie stain (Thermo Fisher Scientific). Individual bands were excised from the gel for LC/MS/MS analysis as described in the Methods. Protein molecular mass standards (Benchmark®, Invitrogen) are in lane 1.

1 2 3


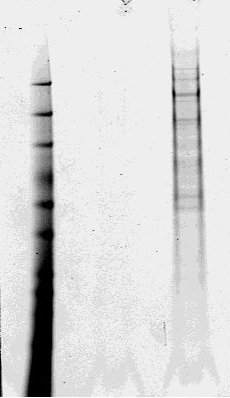


**Supplementary Fig. S3. Antigens immunoprecipitated from Kelly neuroblastoma cells differential detergent extraction** **fraction 2.** Immunoprecipitation was performed as described in the Methods from Kelly neuroblastoma cell differential detergent fraction 2 using an irrelevant IgG3 mAb 14C7 (lane 2), anti-dPSA mAb SEAM 2 (lane 3). Proteins in the samples were resolved on 4–12% gradient SDS-PAGE gels (NuPAGE, Thermo Fisher Scientific). The gels were stained with SimplyBlue™ Coomassie stain (Thermo Fisher Scientific). Individual bands were excised from the gel for LC/MS/MS analysis as described in the Methods. Protein molecular mass standards (Benchmark®, Invitrogen) are in lane 1.

1 2 3 4 5


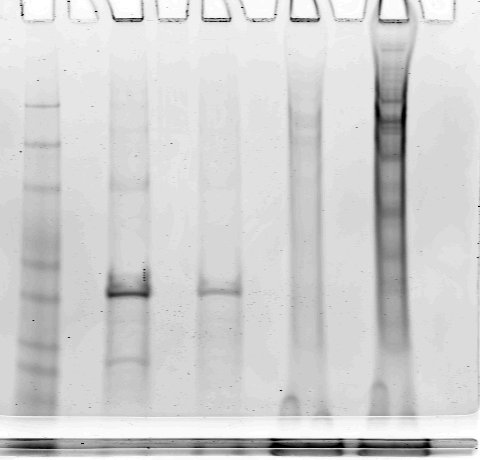


**Supplementary Fig. S4. Antigens immunoprecipitated from PBMCs and SNU-1 gastric cancer cells differential detergent extraction fraction 2.** Immunoprecipitation was performed as described in the Methods from PBMCs (lanes 2 and 4) or SNU-1 cells (lanes 3 and 5) using an irrelevant protein, Neisserial Factor H binding protein (lanes 2 and 3), or anti-dPSA mAb SEAM 2 (lanes 4 and 5). Proteins in the samples were resolved on 4–12% gradient SDS-PAGE gels (NuPAGE, Thermo Fisher Scientific). The gels were stained with SimplyBlue™ Coomassie stain (Thermo Fisher Scientific). Individual bands were excised from the gel for LC/MS/MS analysis as described in the Methods. Protein molecular mass standards (Benchmark®, Invitrogen) are in lane 1.

**Supplementary Fig. S5. dPSA ELISA with nucleolin and SEAM 3**

ELISA with dPSA absorbed to the plate to compare binding of nucleolin with anti-dPSA antibody SEAM 3. The initial concentration of full length nucleolin was based on assumptions of 1 million molecules of nucleolin on the surface of the cell and 10 million cells extracted with detergent in a final volume of 1mL (~10nM). Bound nucleolin was marked with anti-nucleolin antibody MS-3 and dPSA with anti-dPSA antibody SEAM 3. The bound antibodies were detected with alkaline phosphatase-conjugated goat anti-mouse (H+L) secondary antibody (Jackson ImmunoResearch). The ELISA was developed for 60 minutes with substrate and the absorbance at 405nm was recorded as described in the Materials and Methods.

______CHP-134 ____ ____ST8SIA2 KO___ ST8SIA2+ST8SIA4 KO

F1 F2 F3 F4 F1 F2 F3 F4 F1 F2 F3 F4


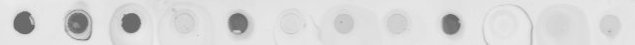


S**upplementary Fig. S6. Immunodot blot of CHP-134, ST8SIA2 and ST8SIA2+ST8SIA4 CRISPR knockout cell line differential detergent extraction fractions**. Portions of each detergent extract fraction from cells prepared as described in Methods were spotted onto a PVDF membrane (Immobilon-FL, Millipore) mounted on a dot blot apparatus (Topac). After blocking with 5% whole dry milk in DPBS blocking buffer, the section of the membrane was incubated with anti-dPSA mAb SEAM 3 (5µg/mL) for 1 hr at ambient temperature in the same blocking buffer and washed with DPBS containing 0.1% Tween 20 three times for 5 minutes. mAbs binding to the membrane were detected with IRDye®800CW Donkey anti-Mouse IgG (H+L) secondary antibody (LI-COR) using the same method as the primary mAb and images were recorded on an Odyssey® Fc Imaging System (LI-COR). The experiment shows that staining in the cytoplasmic fraction (F1) does not depend on either of the polysialyl transferases.
