# Supplementary material for: A cancer-unique glycan: de-N-acetyl polysialic acid (dPSA) linked to cell surface nucleolin depends on re-expression of the fetal polysialyltransferase ST8SIA2 gene
Source: J Exp Clin Cancer Res. 2021 Sep 20;40:293. doi: 10.1186/s13046-021-02099-y (PMC8451149; doi:10.1186/s13046-021-02099-y)
Supplement: Supplementary file 3 — Additional file 3: Supplementary Fig. S7. Human peripheral blood mononuclear cells do not express cell-surface dPSA or nucleolin. Supplementary Fig. S8. Primary and metastatic gastric cancer cell lines express cell-surface dPSA and nucleolin. Supplementary Fig. S9. Primary and metastatic ovarian cancer cell lines express cell-surface dPSA and nucleolin. Supplementary Fig. S10. Primary and metastatic pancreatic cancer cell lines express cell-surface dPSA and nucleolin. [file 13046_2021_2099_MOESM3_ESM.docx]

**Additional File 3: Supplementary Figs. S6–S9.**

**
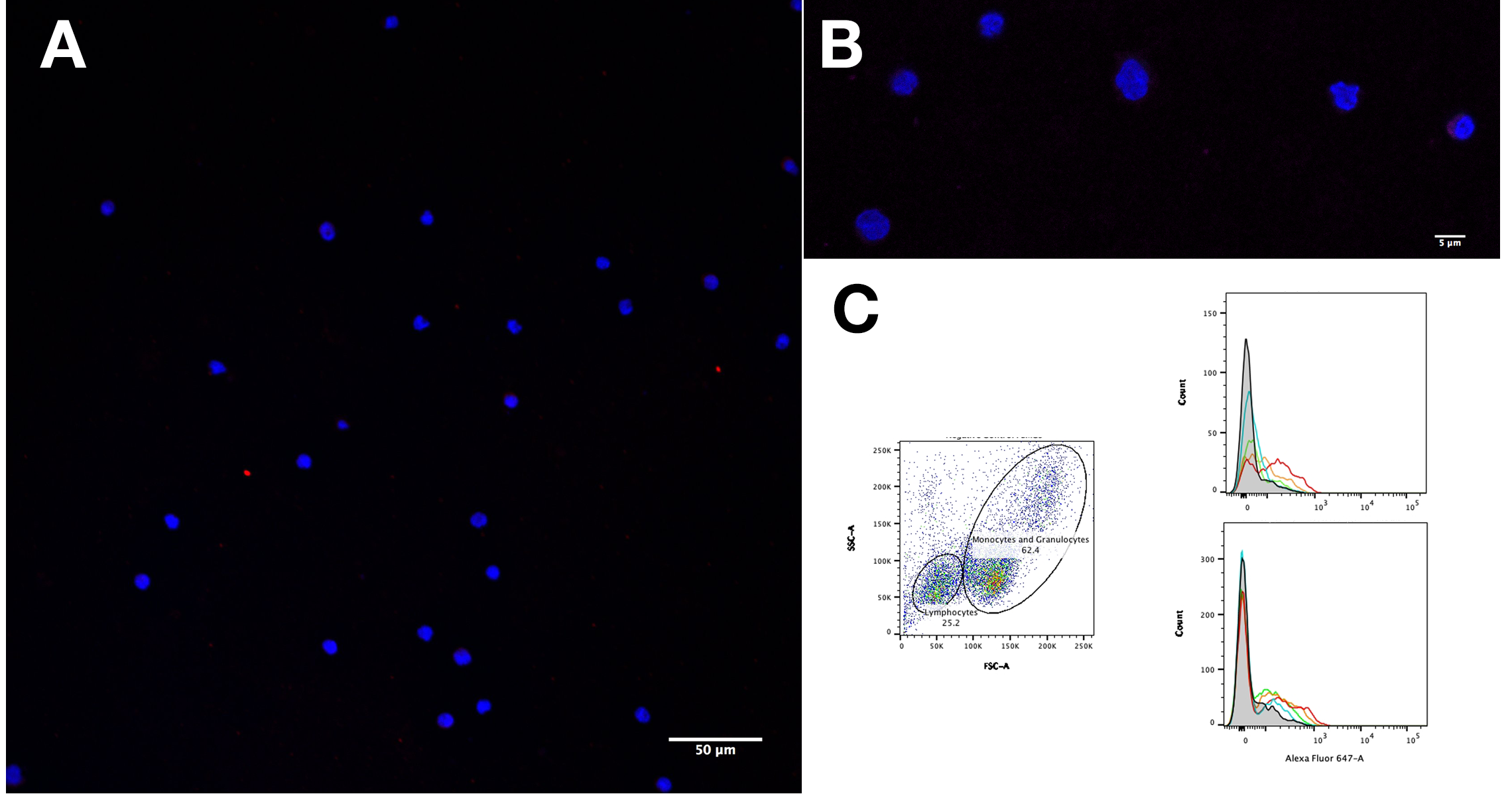
Supplementary Fig. S7. Normal human PBMCs do not have cell surface dPSA.** The possible presence of dPSA was identified on the surface of PBMCs by fluorescently labeling with anti-dPSA antibody SEAM 3 and AlexaFluor 594 (red fluorescence) -conjugated anti-mouse subtype-specific antibody using confocal laser scanning microscopy and by flow cytometry with SEAM 3 and AlexaFluor 647-conjugated anti-mouse subtype-specific antibody. A, 20x magnification. B, 63x magnification. Blue fluorescence is DAPI DNA staining. Scale bars, 50 µm (A) or 5 µm (B). C, SEAM 3 binding by flow cytometry was tested at 10 µg/mL (red line), 3 µg/mL (orange line), and 1 µg/mL (green line). Controls included secondary antibody alone (gray fill) and irrelevant mouse IgG2b mAb from BioXCell (cyan line).

**
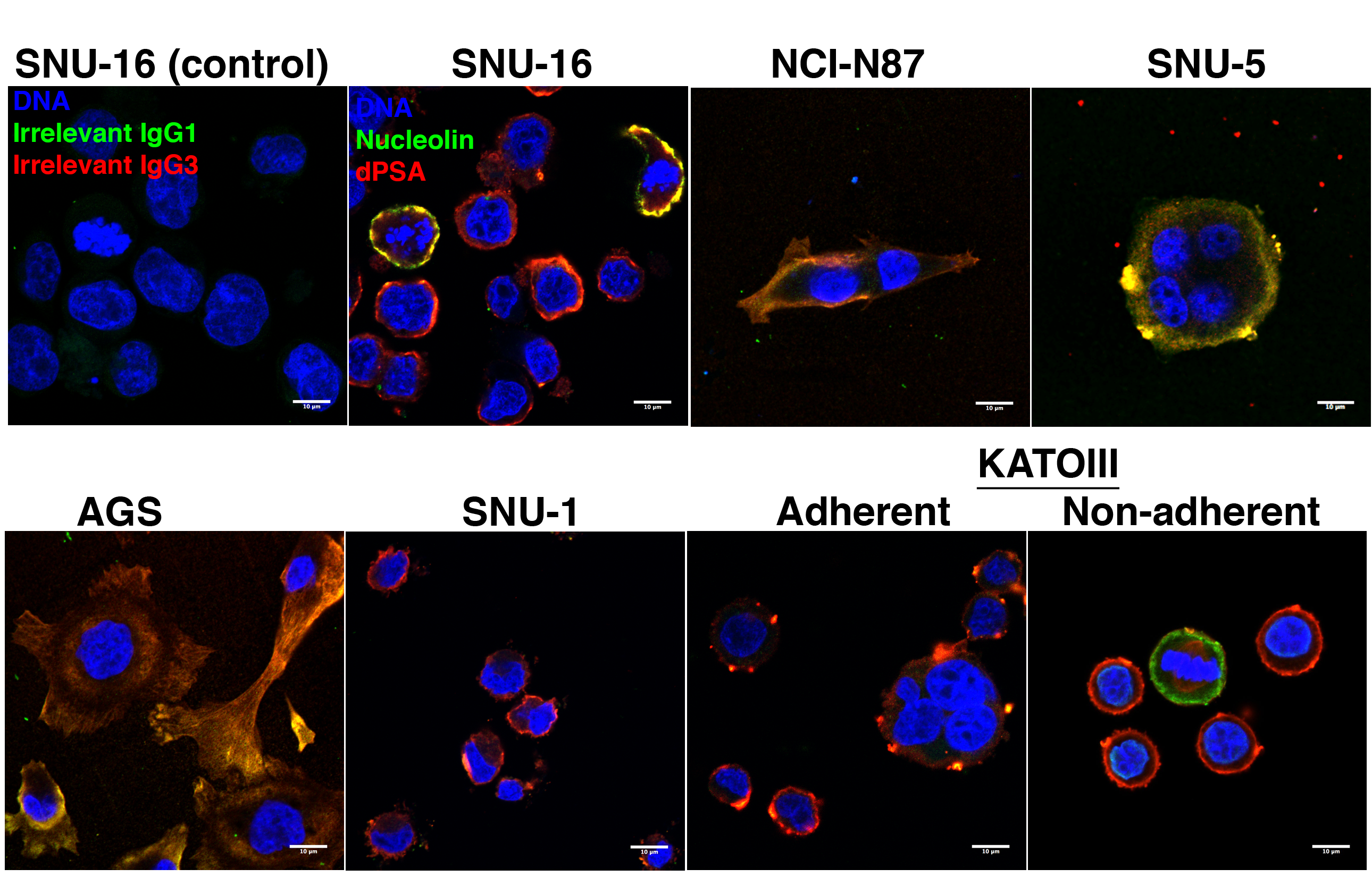
Supplementary Fig. S8. Primary and metastatic gastric cancer cell lines express cell surface dPSA and nucleolin.** dPSA and nucleolin were identified on the surface of cancer cells SNU-16, NCI-N87, SNU-5, and KATOIII from metastatic and AGS and SNU-1 from primary gastric tumors with anti-dPSA mAb SEAM 2 or anti-nucleolin mAb MS-3 and detected by fluorescently labeling with AlexaFluor 594 (red fluorescence) or AlexaFluor 488 (green fluorescence)-conjugated anti-mouse subtype-specific antibodies, respectively, using confocal laser scanning microscopy. A representative example of a cell line (SNU-16) treated with irrelevant mouse IgG1 and IgG3 mAbs and AlexaFluor-conjugated secondary antibodies as a negative control is also shown. Blue fluorescence is DAPI DNA staining. Also shown is reactivity of anti-dPSA and -nucleolin with non-adherent KATOIII cells treated with Triton X-100 to show the presence of dPSA-nucleolin on microtubules of a dividing cell in anaphase I and the movement of nuclear nucleolin to the cell membrane upon dissolution of the nuclear membrane. Scale bars, 10 µm.

**
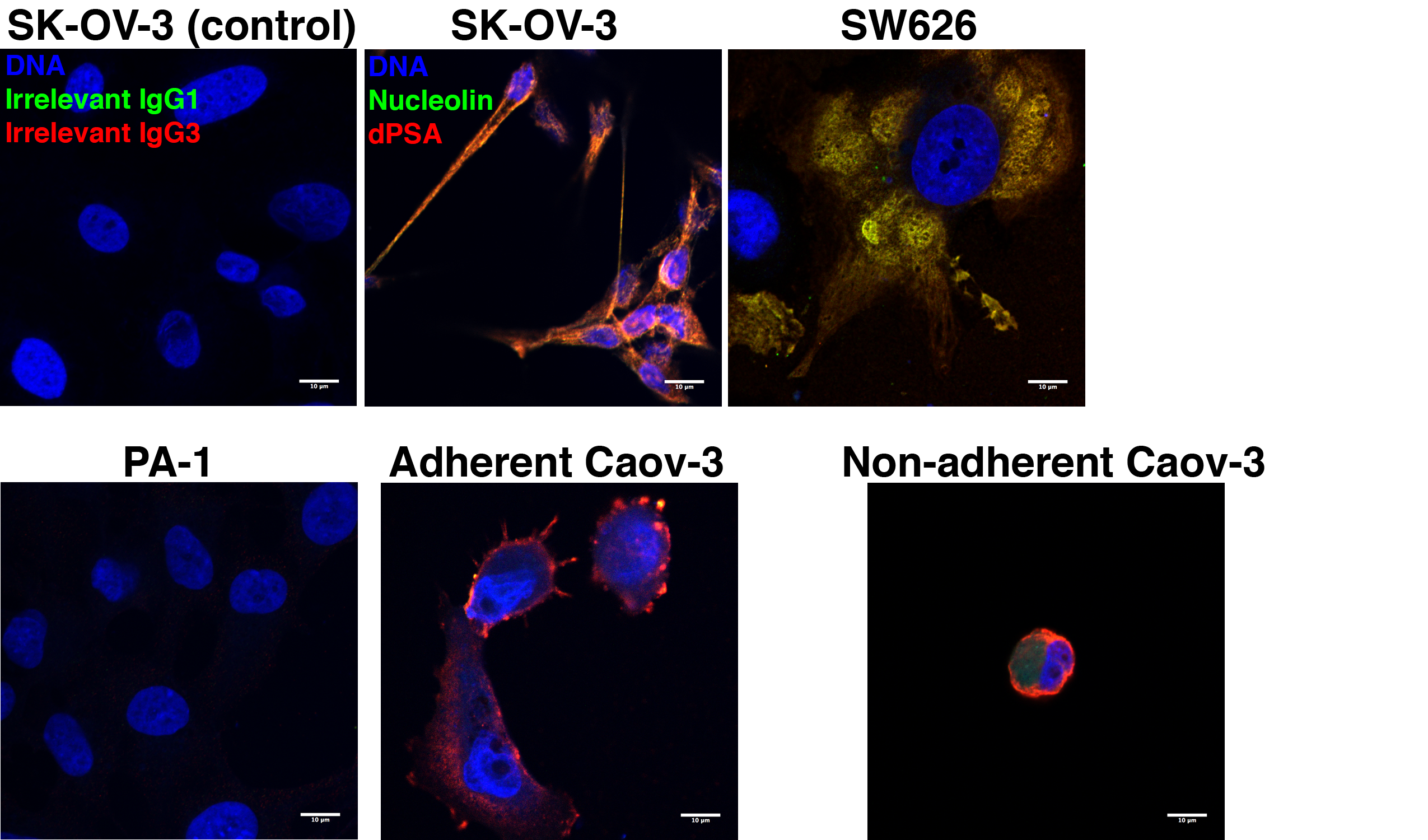
Supplementary Fig. S9. Primary and metastatic ovarian cancer cell lines express cell surface dPSA and nucleolin.** dPSA and nucleolin were identified on the surface of SK-OV-3 from metastatic and SW626 and Cavo-3 (adherent and non-adherent) from primary ovarian tumors with anti-dPSA mAb SEAM 2 or anti-nucleolin mAb MS-3 and detected by fluorescently labeling with AlexaFluor 594 (red fluorescence) or AlexaFluor 488 (green fluorescence)-conjugated anti-mouse subtype-specific antibodies, respectively, using confocal laser scanning microscopy. A representative example of a cell line (SK-OV-3) treated with irrelevant mouse IgG1 and IgG3 mAbs and AlexaFluor-conjugated secondary antibodies as a negative control is also shown. The ovarian teratocarinoma cell line PA-1 was negative for both dPSA and nucleolin. Blue fluorescence is DAPI DNA staining. Scale bars, 10 µm.

**
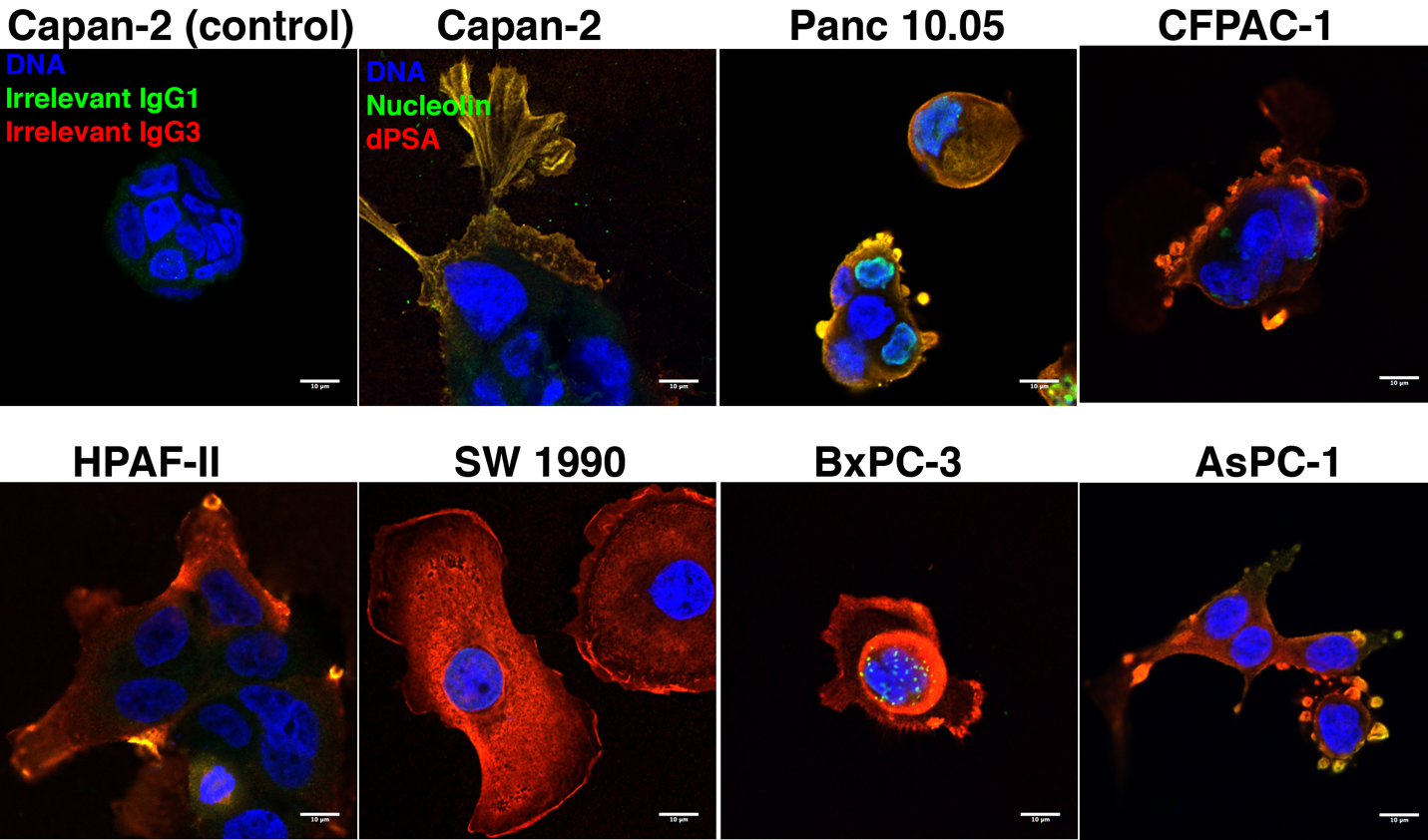
Supplementary Fig. S10. Primary and metastatic pancreatic cancer cell lines express cell surface dPSA and nucleolin.** dPSA and nucleolin were identified on the surface of cancer cells CFPAC-1, HPAF-II, SW 1990, and AsPC-1 from metastatic and Capan-2, Panc 10.5, and BxPC-3 from primary pancreatic tumors with anti-dPSA mAb SEAM 2 or anti-nucleolin mAb MS-3 and detected by fluorescently labeling with AlexaFluor 594 (red fluorescence) or AlexaFluor 488 (green fluorescence)-conjugated anti-mouse subtype-specific antibodies, respectively, using confocal laser scanning microscopy. A representative example of a cell line (Capan-2) treated with irrelevant mouse IgG1 and IgG3 mAbs and AlexaFluor-conjugated secondary antibodies as a negative control is also shown. Blue fluorescence is DAPI DNA staining. Scale bars, 10 µm.
